# Prebiotics and Probiotics Supplementation in Pigs as a Model for Human Gut Health and Disease

**DOI:** 10.3390/biom15050665

**Published:** 2025-05-03

**Authors:** Raffaella Rossi, Edda Mainardi

**Affiliations:** Department of Veterinary Medicine and Animal Science, Università Degli Studi di Milano, Via Dell’Università 6, 26900 Lodi, Italy; raffaella.rossi@unimi.it

**Keywords:** animal model, dietary supplement gut health, pig, human, prebiotic, probiotic

## Abstract

Animal models are an essential part of translational research for the purpose of improving human health. The pig is a potential human research model that can be used to assess the effects of dietary interventions, pathologies, and drugs on gut health and the microbiome, due to its anatomical and physiological similarity to humans. It is recognised that a healthy gut is closely linked to the prevention of several chronic diseases, including obesity, diabetes, gastrointestinal inflammation, as well as neurological and cardiovascular diseases. The use of prebiotics and probiotics plays an important role in maintaining a healthy digestive system, which is responsible for modulating all other body functions. The present review focuses on the applications of prebiotics and probiotics in the pig as an animal model in healthy and diseased conditions, in order to highlight the efficacy of these molecules in the perspective of human health outcomes. The data support the use of prebiotics to improve intestinal health in both healthy and diseased states. In addition, the use of human microbiota-associated (HMA) gnotobiotic pigs provided a good model to study the intestinal and systemic immune response and microbiota composition following probiotic supplementation after a vaccine or virus challenge.

## 1. Animal Model for Human Health

Animal models are essential for translational research aimed at improving human health [1]. In human clinical trials for gut health, there are several confounding factors that can affect the results, such as individual genetic variability and the many factors that influence gut health, including health, lifestyle, and diet [2]. For these reasons, animal models are the first step in evaluating the efficacy of drug and dietary interventions to advance scientific knowledge. Several animal models will be used to study the onset of pathologies in order to develop new therapies to improve human health. Non-human primates (NHP) remain important research models in many biomedical areas, such as infectious disease, immunology, behavioural research, and neuroscience research. They share many similarities with humans in genetic features, anatomy, physiology, and behaviour. Even if it offers an important model for the study of diet-microbiome interactions, there has been very limited use for scientific purposes for ethical and welfare reasons [3]. In fact, it is reported that NHPs account for only 0.28% of the animals used in biomedical research, due to the requirements for their care, the limited number of animals, and the long generation time [4].

Rodent models, in particular murine models, provide many opportunities compared to other species. The availability of genetically modified lines facilitates research aimed at elucidating the interaction between gut health and the onset of disease. The use of the mouse model has some limitations, in particular due to differences in the anatomy of the mouse and human gastrointestinal (GI) tract which may be influenced by their different diets, feeding patterns, body sizes, and metabolic requirements [5]. In particular, there is a non-glandular forestomach, which is used for food storage and is covered with a biofilm made up of different strains of *Lactobacillus* spp., followed by a glandular stomach. The feeding behaviour, pH range of the stomach, and the presence of different bacterial strains are influenced by these anatomical differences [6]. Moreover, the length of the small intestine per kg in humans is 10 cm/kg and in mice 1500 cm/kg and the large intestine is more developed in mice. These differences affect intestinal transit time, which may influence the intestinal microbiota [5]. Moreover, mice injury models fail to replicate human disease [7].

A potential human research model that can be used to assess the effects of dietary interventions, pathologies, and drugs on gut health and the microbiome is the pig, given the importance of these last two factors in the pig farming system [8]. Although more expensive to use in clinical trials, pigs have been used for many years as biomedical models because of their anatomical and physiological similarities to humans, making them an ideal model for research [7]. Moreover, pigs are omnivores, have nutritional requirements comparable to humans, and exhibit analogous metabolic and intestinal physiological mechanisms [9,10]. The sequencing of the complete genome of the domestic pig has increased the potential applications of this animal model [11]. In fact, their gene regulation mechanisms were similar to humans [12].

The gut anatomical similarities to humans contribute to analogous transit time and digestion and absorption processes [7]. In both species, also the molecular mechanisms and taste perception are similar for sweet, umami, sour, and fatty acid tastes, with some differences for salty and bitter tastes. In the regulation of satiety, the plasma concentrations of cholecystokinin and glucagon-like peptide-1 are comparable in pigs and humans, whereas some differences were observed for peptide YY (PYY) and ghrelin levels between the two species [13]. This makes the pig model a suitable candidate for the study of the effects of dietary interventions on taste perception and the regulatory mechanisms that modulate appetite and satiety [14]. Vitamin and mineral intake in human and swine diets are similar during infancy, growth, reproduction, and lactation [14]. This contributes to their comparable mucosal barrier and microbiota physiology and susceptibility to certain pathogens [15,16]. The morphological characteristics of the intestine such as the structure of the villi, the cells of the intestinal epithelium such as the stem cells, goblet cells, and enteroendocrine cells as well as the enterocytes are also similar to those of humans. Therefore, some comparisons in the gut microbial ecology between pigs and humans have made the pig a suitable model for studies of how dietary intervention can modulate gut health [8]. In fact, in both species, *Firmicutes* and *Bacteroidetes* are the main phyla of the gut microbiota [8]. Table 1 highlights the importance of the pig as an animal model for the study of human health and disease.

The use of porcine models presents some challenges related to high costs and the limited availability of specialised facilities. Indeed, the care of pigs is considerably more expensive, as they require specialised facilities, space, ventilation, and trained personnel for daily management. Genetics is also a potential issue, as pigs have a more complex genome than other laboratory animals, such as mice, which limits the possibilities for genetic manipulation [24].

There are also several challenges that need to be addressed when using the pig as a model for humans. In fact, the translation of results may be affected by differences in the composition of the microbiota, in particular the greater presence of *Bifidobacteria* in the human intestinal tract than in the pig [25]. As reported below, the use of human microbiota-associated (HMA) gnotobiotic pigs provides an improved model to study human gut health [18]. In particular, the use of the HMA gnotobiotic porcine model has great potential to mitigate some difficulties related to studying host–microbiome interaction in both animals and humans. Moreover, animal models with ‘humanised’ microbiota have been created inoculating animals with microorganisms isolated from the human gut. These piglets defined as HMA have a microbiota much closer to that of humans than that of conventionally reared piglets. Pigs, in particular, given their similarities to the human gut, have been the animals most involved in this research. Piglets associated with the human microbiota were therefore created using inocula from infants, children, and adults [26]. It is reported that a high percentage of taxa from humans are able to colonise and persist in the GI tract of HMA piglets [27].

This animal model has significantly facilitated the progress of research into the physiology, metabolism, and immunity of the human gut. There are already some studies showing the results obtained using these animals. In a review of Vlasvoa, the main studies using HMA piglets were analysed. In particular, it was reported how useful these animals can be for evaluating potential nutritional interventions to contrast the effects of malnutrition and dysbiosis [18].

It is also to consider the variability in the disease model, in fact, enterotoxigenic E. coli (ETEC) challenge models in pig and human inflammatory bowel disease (IBD) models may vary due to genetic plasticity, differences in challenge dose and strain, and other factors as reported by Mirsepasi-Lauridsen (2019) [28]. It is also important to consider the appropriate translation of the dose, and indeed there are some methods used in experimental research to predict an approximate dose on the basis of existing data in other species [29]. In fact, before being used to improve human health, the dose effects of any products should be fully investigated in clinical trials.

In this review, the differences and similarities between the porcine and human gastrointestinal systems were reported. The topic of the paper is the application of prebiotics and probiotics in the pig as an animal model under health and disease conditions to highlight the efficacy of these molecules in terms of human health outcomes.

## 2. Gastrointestinal Tract

The GI tract includes the oral cavity, pharynx, oesophagus, stomach, small and large intestines, and the accessory structures that make up the digestive system [30]. The GI tract can affect the digestion and absorption of nutrients, secretion, and excretion of by-products of digestion and it also performs physiological functions essential to overall health. Recent studies have shown that a healthy GI tract is closely linked to the prevention of several chronic diseases, including obesity, diabetes, gastrointestinal inflammatory conditions, as well as neurological and cardiovascular disease [31]. In fact, changes in the morphology and physiology of the GI tract are associated with several diseases [32]. In particular, the several components of the intestinal tract, the gut microbiome, and the host immune system are all involved in intestinal homeostasis [33]. The intestinal tract composed of the small intestine and large intestine is in constant contact with the external environment and all exogenous factors. Some differences between the human and pig intestinal tract are related to their length and the spatial arrangement of the intestine within the abdominal cavity. However, the ratio of intestinal length per kilogramme of body weight is comparable in the two species [15]. The anatomical and morphological characteristics of the gastrointestinal tract of pigs are similar to those of humans. However, the distinction between the duodenum, jejunum, and ileum in the porcine small intestine is less pronounced than in humans. Furthermore, the digestive and metabolic processes, function, and digesta transit time are comparable to those of humans [7]. The dynamic state of maintenance of healthy intestinal homeostasis and inhibition of inflammation requires interactive relationships among the intestinal microbiota, the intestinal epithelium, and the immune system [34].

### 2.1. Intestinal Barrier

The mucosa is a highly selective barrier that protects against pathogens and facilitates the absorption of nutrients [35]. The intestinal barrier not only acts as a physical barrier that prevents harmful substances including microorganisms, antigens, and proinflammatory factors from entering the body, but also acts as a functional carrier, allowing nutrients and drugs to be absorbed, metabolised, and transported across the body [36].

The intestinal epithelial cells (IECs) are responsible for the generation of physical and chemical barriers, which are essential for the protection of the intestinal mucosa from commensal microbes and invading pathogenic microorganisms [37]. The physical barriers encompass the mucus layer, the glycocalyx, which is located on the microvilli of absorptive intestinal epithelial cells, and the cell junctions, which are responsible for firmly linking intestinal epithelial cells. The chemical barriers comprise antimicrobial proteins (AMPs) and the proteins regenerating islet-derived 3 (Reg3), secreted by Paneth cells. These barriers act as a physical impediment to the invasion of the mucosa by intestinal microorganisms [38].

The intestinal barrier consists of three different layers: the inner layer, called lamina propria, of innate and adaptive immune cells, the middle layer of IECs, and the mucosal layer of epithelial cells, AMPs, secretory immunoglobulin A (sIgA), and the intestinal microbiota [39]. Furthermore, the intestinal lumen is covered by a mucus layer, constituted by highly glycosylated mucin proteins, which serves to limit any interaction between microorganisms and epithelial cells. A key determinant of intestinal barrier function is a group of proteins that seal the space between adjacent cells near the apical membrane, defined as a tight junction. Claudins are recognised as fundamental constituents of tight junctions, which are involved in the modulation of the paracellular permeability of ions and molecules. Occludin may be involved in tight junction arrangements with the support of scaffolding proteins, such as zonula occludens. Tight junction defects are potentially responsible for the impairment of the intestinal barrier and the development and progression of GI diseases [40].

#### 2.1.1. Mucus Layer

In the intestinal tract, the predominant component of the mucin layer is MUC2 and trefoil factor 3 (TFF3), which are produced by goblet cells [41,42]. Alterations in mucus composition or production have been linked to the development of gastrointestinal diseases [43]. In addition to its barrier function, mucus facilitates the passage of food along the digestive tract, preventing the direct contact of potentially harmful agents with the epithelium. Furthermore, mucins play a dynamic role in signalling and modulating the immune response within the gastrointestinal tract. The interaction between mucins and the immune system helps in the detection and neutralisation of pathogens, thereby contributing to the overall immune defence. The small intestinal mucus mixes with Paneth cell secretion containing antibacterial peptides, and lysozyme. It has been demonstrated that Paneth cell products, combined with antibacterial proteins produced by enterocytes, generate an antibacterial gradient in mucus, thereby preventing bacterial adhesion to epithelial cell surfaces [44].

It has been observed that the large intestine exhibits a distinct mucus organisation compared to the small intestine, which features a two-layered system. The inner mucus layer contains polymerized MUC2, which is attached to the surface of the intestinal epithelium and gradually expands outwards, forming an outer layer that continuously secretes mucus and prevents the easy invasion of the intestinal epithelium by microorganisms [42]. The outer layer of mucus, on the other hand, is colonised by a large number of gut bacteria that use the polysaccharides of MUC2 as an energy source, thereby providing nutrients and adhesion sites for symbiotic flora [43]. It has been demonstrated that there is a substantial absorption of short fatty acids by tissue epithelial cells; moreover, butyrate has been shown to play a pivotal role in the protection of colon epithelial cells and metabolism [30]. In addition, it has been demonstrated that bacterial metabolites can affect the host immune system, host metabolism, and, more recently, host brain function [45]. The inner mucus layer is ordinarily effective in preventing the penetration of bacteria; however, some microorganisms have developed mechanisms to overcome this defence system by secreting proteases [46].

In a recent study, a comparable microstructural organisation of the mucus in relation to the permeability to particles was observed in adult pigs and humans and this supports the use of small intestine porcine mucus to effectively mimic the transport of nutritional supplements or drugs in humans [47].

#### 2.1.2. Intestinal Epithelial Cells

The IECs, a single layer of cells organised into crypts and villi, cover an area of ~400 m^2^, making it the largest mucosal surface in the body [48]. The epithelial cells constitute a layer of cells that form a functional barrier to protect the intestinal mucosa from commensal or invasive pathogens. These cells are generated by stem cells found in the crypts and replaced continuously, at a rate of 4–5 days in humans and about 7 days in pigs, through a process of proliferation and migration. It is well established that the intestinal epithelium comprises several cell types that can be divided into the following: secretory and absorptive. Moreover, the tuft cells are chemosensory cell types found in the gastrointestinal tract. Tuft cells (TCs) are epithelial cells distinguished by the presence of a tubulovesicular system and an apical bundle of microfilaments, which are connected to a tuft of long and thick microvilli protruding into the lumen [49]. These cells, which are found in many tissues such as the thymus, pancreas, urethra, and GI tract play a central role in the body’s defence against helminth or protozoan infections by activating type 2 immunity [50].

##### Secretory Cells

Two important intestinal secretory cell types are Goblet and Paneth cells, with distinct but complementary functions for gut health. Goblet cells represent the most common type of secretory cells in the intestinal tract [51]. They derive from stem cells located near the base of the intestinal crypts and contain vesicles containing proteins such as MUC2, β-molecule resistin-like, and Fc-γ-binding protein [52]. Their main role is to protect the intestinal epithelium thanks to the mucus layer they create. Paneth cells, on the other hand, located in Lieberkühn’s crypts, that are small invaginations lining the surface of the mucosa, secrete AMP-rich granules such as lysozyme and various antibacterial proteins such as α-defensins, lysozymes, secretory phospholipase A2, angiogenin-4, RegIIIγ, RegIIIβ and α1-antitrypsin that help defend the host against infection and maintain a balanced microbiota [53]. They also play a crucial role in innate immunity and are involved in stem cell proliferation and thus in the turnover of the intestinal epithelium [54]. As essential components of the intestinal system, Goblet and Paneth cells play a key role in defending against external pathogens and ensuring the stability of the epithelial environment.

Enteroendocrine cells (EECs), which make up 1% of the total intestinal epithelial cell population, secrete hormones and peptides in response to luminal contents and regulate metabolism by coordinating digestion, absorption, nutrient excretion, and satiety [55]. The EECs can have an effect on neighbouring cells and on distinct neuronal pathways including enteric and extrinsic neurons [56]. The EECs are distinguished by their capacity to detect a broad spectrum of substances within the lumen, encompassing nutrients, non-nutrient chemicals, food-borne toxins, and microorganisms. When stimulated, the EECs secrete signalling molecules that activate neural circuits, which in turn trigger the transmission of information to different regions of the brain, thereby inducing the appropriate functional responses [57]. This process results in the regulation of several functions, including motility, inflammatory response, immunity, and blood flow [52]. Therefore, ECCs have the ability to coordinate gut responses to nutrient introduction through the secretion of hormones/peptides that induce gastrointestinal, pancreatic, and biliary responses, as well as the modulation of GI motility. Potentially harmful substances in the lumen can induce a protective response to decrease or avoid the potentially harmful effects by delaying gastric emptying, increasing intestinal secretions, as well as inducing vomiting and diarrhoea.

##### Absorptive Cells: Enterocytes and Colonocytes

The intestinal epithelial tissue is predominantly composed of enterocytes, accounting for approximately 80% of the total intestinal epithelial cells [58]. These cells are characterised by their columnar morphology and are bound to surrounding cells by tight junctions. These cells are distinguished by the presence of microvilli on their apical membranes, with a density ranging from 3000 to 7000 per cell. The enterocytes in the small intestine exhibit a threefold increase in microvilli compared to those found in the colon (called colonocytes). This increased microvillus density in enterocytes of the small intestine is thought to contribute to an augmented absorptive surface area, thereby enhancing their capacity for nutrient absorption [59]. The apical tips of the microvilli are characterised by the presence of large, negatively charged, integral membrane glycoproteins similar to mucins. This approximately 400–500 nm-thick layer is known to contain adsorbed pancreatic enzymes and intramembrane glycoprotein enzymes that are responsible for the process of terminal digestion This thick layer has been observed to act as a protective barrier against particles, viruses, and/or bacteria by preventing the uptake of antigens and pathogens, thus providing protection from disease. It also provides a highly degradative microenvironment that promotes the digestion and absorption of nutrients [60].

### 2.2. Microbiota

The gut microbiota is defined as a complex community of microorganisms, including bacteria, viruses, fungi, and protozoa, that populate the gastrointestinal tract [61]. The gut microbiota is made up of four main phyla, including *Firmicutes*, *Bacteroidetes*, *Actinobacteria,* and *Proteobacteria*, with *Firmicutes* and *Bacteroidetes* making up 90% of the gut microbiota [62]. The composition of the gut microbiota differs for each individual and is affected by diet, lifestyle, the drugs or antibiotics used, and age. In fact, the composition of the microbiota is shaped by various external host and environmental factors and changes with age, with a gradual increase in microbial diversity during early life and relative stability during development and adult life [63]. The microbiota provides multiple benefits to the host influencing several physiological processes such as digestion, supporting the absorption of nutrients and/or the fermentation of some molecules that produce important metabolites, including short-chain fatty acid (SCFA), immune homeostasis, and act as a barrier against pathogens by colonising the mucosa in order to remove nutrients from pathogens and producing antimicrobial substances [64]. It may affect the synthesis of essential vitamins, in particular, vitamin K and most water-soluble B-vitamins such as thiamine (B1), riboflavin (B2), niacin (B3), and pantothenic acid (B5) [65]. Moreover, it is widely acknowledged that the gut and brain are in constant bidirectional communication, of which the gut microbiota and its metabolic production are a major component, forming the so-called gut microbiome–brain axis [66]. Similar to humans, the gut microbiota of pigs primarily comprises the *Firmicutes* and *Bacteroidetes* phyla. Although there is a clear similarity between the composition of the human and pig microbiota at the phylum level, with both having predominantly *Firmiticutes* and *Bacteroidetes*, there are substantial differences at the species and genus levels. In the pig GI tract, the key bacterial groups are: *Streptococcus* spp., *Lactobacillus* spp., *Eubacterium* spp., *Fusobacterium* spp., *Bacteroides* spp., *Peptostreptococcus* spp., *Bifidobacterium* spp., *Selenomonas* spp., *Clostridium* spp., *Butyrivibrio* spp., *Escherichia* spp., *Prevotella* and *Ruminococcus* spp. [8], while humans more commonly have genera such as *Bacteroides*, *Faecalibacterium*, *Roseburia*, and *Bifidobacterium* [67]. These differences are due to the substantial differences in lifestyle and dietary habits between humans and animals. In particular, the balance between *Bacteroides* and *Prevotella*, two genera whose relative abundance is often associated with dietary patterns. The first, in fact, dominates in humans whose dietary habits are rich in fat and protein; the second, on the other hand, is especially part of the animal model as diets are plant-based and high in fiber, a detail that marks another difference [25]. In fact, with a diet richer in fiber, the pig microbiota has more anaerobic bacteria. Furthermore, Bifidobacterium, a gene that is considered beneficial and widely used as a probiotic in humans, is much more abundant in the human gut than in pigs, where it is found only in small amounts; similarly, *Akkermansia muciniphila*, a genus associated with metabolic and barrier functions in humans, is also scarcely present in the pig gut. RIF. These differences in the microbial population of the microbiota may influence the production of key metabolites such as SCFA, which play crucial roles in intestinal and systemic homeostasis. Knowing these differences allows a better understanding of the evaluation of pig models in studies focusing on the microbiota [12].

## 3. Dietary Intervention

It has been established that diet optimal dietary nutrition exerts a positive influence on the overall health status of the individual affecting numerous pathways, exerting a direct impact on host physiology and indirect impacts on the modulation of the microbiota [68]. In fact, a balanced diet can sustain the immune response by providing essential nutrients that decrease inflammation processes, enhance intestinal barrier integrity and permeability, and modulate the gut–brain axis [69,70]. It is reported that a healthy diet presents a high content of fiber, polyphenols, and unsaturated fatty acids [71]. Some dietary interventions, such as prebiotics and probiotics can support gut health and the overall well-being of both humans and animals [72,73].

## 4. Prebiotics

The definition of a prebiotic was revised in 2017 as “*a substrate that is selectively utilised by host microorganisms conferring a health benefit*”, expanding to include new compounds such as amino acids, nucleotides, and polyphenols [74]. Prebiotics are typically non-digestible carbohydrates that resist digestion in the upper gastrointestinal tract and are fermented in the colon, where they promote the growth and activity of beneficial bacteria [75]. This microbial stimulation leads to increased production of short-chain fatty acids (SCFA), which promote gut health. In this study, we focus on the effects of the main oligosaccharides on gut health. *Oligosaccharides* are classified as carbohydrates that consist of a small number of monosaccharide units (3 to 10) covalently linked by glycosidic bonds [76]. They can be synthesized through different processes: natural derivation, synthesis through the physical, chemical, or biological breakdown of polysaccharides, and the use of microbial cell and enzymatic processes [77]. The most well-known oligosaccharides used as prebiotics are FOS, MOS, and GOS [78].

FOS naturally found in several plants, with a smaller content in cereals [79], promotes *Lactobacillus* and *Bifidobacterium* growth, while reducing *Enterobacteriaceae* [80,81]. This improves gut health and enhances immunity by maintaining an optimal gut microbiome and protecting against enteric diseases [82]. Moreover, FOS stimulates several signalling pathways that play a crucial role in the regulation of tight junctions [83].

MOS is typically derived from yeast cell walls or plant mannans [84,85]. They support probiotic activity by improving host health; in fact, several studies show that MOS promotes microbial populations of *L. reuteri* and *L. salivarius*, *L. brevis*, *L. delbrueckii*, *L. acidophilus*, *L. rhamnosus*, *Bifidobacterium adolescentis*, and *B. animalis* [86,87]. Moreover, they have been shown to promote immune homeostasis and reduce inflammation in vivo and in vitro [83].

GOS gained significant attention due to their prevalence in milk and their role as active substances. They play an important role in the development of the intestinal microbiota and the immune system, especially in the early stages of life [88]. Moreover, it is observed that GOS are able to attenuate the tight junction impairments caused by several stressors (antinutritional and environmental factors). GOS promotes the growth of beneficial bacteria such as *Bifidobacterium* spp., *Lactobacillus* spp., and *Ruminococcus* spp., These phenomena result in increased production of SCFA and the recovery of dysbiotic populations, alongside concurrent suppression of inflammatory activity [89].

In humans, some studies reported that FOS supplementation (5 g/day) for 6 weeks and GOS supplementation (3.5 g/day) for 12 weeks improve symptoms due to irritable bowel disease (IBD) [90,91]. In addition to a study in patients affected with ulcerative colitis (UC), GOS supplementation showed positive effects on the symptoms of the pathology. [92]. A recent review by Zhang et al. reported a positive variation in gut microflora with prebiotic intervention, with higher numbers of *Bifidobacterium* in the GI tract of patients with IBD [93]. It is also observed that in child dietary supplementation with MOS infant formula (7.2 g MOS/L) for 6 months, changes the gut microbiota, enhancing *Bifidobacterium* spp. and faecal pH. A reduction in faecal pathogens and an improvement in intestinal immune response were also observed [94]. In addition, several prebiotic oligosaccharides, have been shown to increase the abundance of Bifidobacteria and Lactobacilli, similar to human milk oligosaccharides (HMA) [95,96].

### Prebiotics in Pigs: A Model for Human Health

It is interesting to assess how dietary integration with prebiotics in pigs can become a model for humans given the physiological and anatomical similarities between them, especially regarding the GI tract [8]. The use of piglets is fundamental to studying therapeutic approaches for the treatment of complex human diseases, which begin in infancy. Indeed, they allow us to study fundamental principles in the areas of nutrition, gut microbiota development and host immune function, as well as pathology such as necrotizing enterocolitis (NEC) and short bowel syndrome (SBS) [97]. It has also been observed that endemic diarrhoea in infants occurs after an ETEC infection with an incidence of 10 to 30% [98]. In addition, it is reported that rotavirus (RV), which is the most important agent for diarrhoea in children, is also one of the most important viral agents responsible for the development of diarrhoea in piglets [8,99]. Moreover, *Salmonellosis* is an important disease in humans and in the world, it is the second cause of bacterial foodborne zoonoses in humans [100].

Many studies are conducted in pig models in order to evidence the effect of dietary supplementation with prebiotics on gut health. A literature search was carried out in CAB Abstracts and PubMed to identify articles published between 2005 and 2025. Further articles were identified by examining the reference lists of these articles. Results were limited to trials in these ranges of the following parameters: use of pigs as human models; dietary supplementation with prebiotics; and healthy or infected animals.

Considering that the main pathogenic bacteria that induce gut disease in humans are *Salmonella* spp., and some strains of *Escherichia coli* [101] some included studies reported the effects of prebiotics on infected animals. The studies were mainly conducted on suckling and weaning piglets, with the aim of establishing the effects of the prebiotics in the early postnatal life of the subjects. The nutritional function of prebiotics was evaluated in piglets under healthy conditions and during pathogen challenge (*Salmonella*; ETEC; RV strain) and data were compared with a challenge control group without dietary supplementation.

The data reported in Table 2 highlight that dietary intervention with GOS and FOS are able to positively affect morphological parameters, such as villus height, the ratio between villus height, and crypt depth ratio, associated with improvement of nutrient adsorption [102,103,104]. Moreover, an improvement in the expression of tight junction proteins (ZO-1, claudin-1, and occludin) in ETEC-challenged animals, and an improvement in epithelial barrier components was also observed. In addition, enhanced SCFA production and positive modulation of the microbiota composition were also observed in healthy and infected animals [84,105].

When lactulose was added to the diet of weaned piglets orally challenged with *Salmonella Typhimurium*, the IgG, IgA, and IgM levels were significantly increased and a lower pathogen shedding compared to the control diet group was also observed [106]. The mechanisms of action of prebiotics on the immune system are not fully understood. It has been hypothesized that they modulate the gut microbiota and enhance SCFA production [104,107,108] and in vitro study reported that mesenteric lymphocytes cultured with SCFA increased the production of both IFN-γ and IL-10 [109].

Another study by Shen et al. showed that HMA piglets could be used as a model for evaluating the effects of short-chain fructo-oligosaccharides (scFOS) on gut health. In fact, the study showed that using HMA piglets, the bifidogenic properties of scFOS were confirmed and it was also found that the effects of scFOS on non-bifidobacteria varied in the different developmental stages of the animals, an interesting result also for evaluating the effects of these supplements in humans [110].

**Table 2:** Experimental studies in pigs fed prebiotics utilising as a human model.

Prebiotic Supplement	Dosage	Length (d)	Animals	Effects in GI Tract	References
Short-chain FOS	1% enteral nutrition	5	Neonatal piglets (2–7 d)	↑ Ileum Villus height **	[102]
Short-chain GOS Long chain FOS	3.6 g/L 0.4 g/L	14	Neonatal Piglets (1–15 d) Porcine RV challenge (10 d)	↓ diarrhoea * ↑ circulating RV IgM **	[111]
Pectic oligosaccharides product	200 mg/kg	18	Weaned Piglets (21–40) Porcine RV challenge (15 d)	↓rate of diarrhoea * ↑ Ileal and jejunal RV antibody ↑ Ileal and jejunal sIgA	[112]
Lactulose	1%	17	Weaned piglets (21–42 d) *Salmonella Typhimurium* challenge (7 d)	↑ IgG, IgA, IgM * ↓ shedding of pathogen *	[106]
MOS (98%)	0.3%	21	Weaned piglets (21–42 d) ETEC challenge (19 d)	↑ ZO-1 expression * ↑ sIgA * ↓ proinflammatory cytokine secretion *	[105]
MOS (20%)	0.06%	21	Weaned piglets (21–42 d) ETEC challenge (19 d)	↑ ZO-1 expression * ↑ claudin-1 expression * ↑ Ileum and duodenum Villus height * ↑ Villus height/crypt depth ratio *	[84]
Short-chain GOS (21%)	milk replacer 0.8% GOS	3 26	Suckling Piglet (1–4 d) Suckling Piglet (1–27 d)	4 days ↑ Duodenum villus height * ↑ Duodenum villus height/crypt depth ratio** 27 days: ↑ *Lactobacillus* ** Bifidobacterium spp. ** ↑ Colon occludin gene expression *	[113]
GOS and polydextrose	0.8% prebiotic mixture (50/50 GOS/PDX)	21	Suckling Piglets (1–22 d)	↑ *Bacteroidetes* and *Firmicutes* phila * ↑ SCFA Concentrations ** ↓colon pH **	[104]
GOS	10 mL of GOS solution daily	7 20	Suckling Piglets (1–8 d) Suckling Piglets (0–21 d)	↑ *Firmicutes* philum * ↑ *Lactobacillus* * ↓ *Streptococcus* and *Clostridium* * ↑ SCFA Concentrations * ↓Ileal pH * ↓ *Bacteroidetes* philum * ↑ *Lactobacillus* * ↓ *Escherichia* spp. *	[108]

PDX, polydextrose; FOS, fructooligosaccharides; ZO-1 zonulala occludins 1, SCFA, short chain fatty acids; GOS, galacto-oligosaccharides; MOS, mannano-oligosaccarides; ETEC, enterotoxigenic escherichia coli; RV, Rotavirus; ↑ increase; ↓ decrease; * *p* < 0.05; ** *p* < 0.01.

These data strengthen the evidence for using prebiotics to support gut health in piglets used as human models in both healthy and diseased conditions. In fact, prebiotic supplementation can affect the gut *Bifidobacterium* and *Lactobacillus* content and restore intestinal barrier function and morphological parameters, reducing the onset of several gastrointestinal diseases in humans [89]. The gap in the reported studies is related to the high heterogeneity of dosage and duration of supplementation, which should be better standardised. In addition, when considering the differences in microbiota, the use of HMA pigs should be considered to obtain more reliable results.

## 5. Probiotics

The term dysbiosis is employed to denote an imbalance in the structure and function of intestinal microorganisms [114]. This condition arises from various factors, including bacterial infections, antibiotic use, and alterations in dietary habits. The utilisation of probiotics constitutes a pivotal component in the process of restoring healthy commensal microbial communities [115]. Probiotics are defined as “*live microorganisms which when administered in adequate amounts confer a health benefit on the host*” [116]. In order to fulfil their primary functions, probiotics must demonstrate the capacity to survive and proliferate within the intestinal tract. Here, they can influence the populations and activities of microbiota that colonise this tract. At the GI level, they promote an improvement of the intestinal barrier by stimulating mucin production, regulating tight junction expression, and promoting the intestinal immune response. Finally, they also produce antimicrobial substances such as SCFA, organic acids, and hydrogen peroxide, which promote resistance against microbial agents [117]. The ability of certain probiotics to protect the host against these toxins is responsible for their efficacy in treating diarrhoea. In fact, toxins are probably the most important group of virulence factors in bacteria [118].

Moreover, probiotics can perform also other several positive effects on the human organism. Indeed, it has been demonstrated that they are able to compete with pathogens for nutrients and adhesion sites on the GI mucosa, exerting antimicrobial activities [119].

There is a wide range of microorganisms that have been studied as probiotics, resulting in many commercial products that are promoted and marketed as food supplements for humans or feed additives for animals [119]. The probiotic product’s composition varies from single-species/strain to multispecies/strain microorganisms. The most commonly used probiotics belong to the following genera: *Lactobacillus*, *Bifidobacterium*, *Bacillus*, and *Enterococcus Streptococcus.* It is also ustilized yeast belonging to the genus *Saccharomyces* [120]. The *Lactobacillus* and *Bifidobacterium* spp. are prominent genera within the human gut microbiota and have become the most used probiotics [121].

The description of genera and strains used as supplements in humans is reported by Sarita et al. [122]. *Lactobacillus* spp. are Gram-positive bacteria that produce lactic acid in the GI tract. They are anaerobes that can improve the uptake and bioavailability of minerals and reduce intestinal permeability. They are anaerobic bacteria that improve mineral absorption and bioavailability and lessen intestinal permeability. The common *Lactobacillus* strains used as probiotics are the *acidophilus*, *rhamnosus*, *fermentum*, *johnsonii*, *lactis,* and *reuteri. Bifidobacterium* are Gram-positive, anaerobic bacilli that generate acetic acid and lactic acid as metabolic by-products. The common *Bifidobacterium* strains used as probiotics are the *infantis*, *longum*, *bifidum*, *lactis,* and *thermophilum. Bacillus coagulans*, even if is not present in normal intestinal flora, produce lactic acid, preventing pathogen colonisation and restoring GI tract microbiota. In addition, *Saccharomyces cerevisiae* is commonly used for restoring the dysbiosis caused by diarrhoea. Probiotic supplements have been used to treat GI tract disorders such as infectious diarrhoea, antibiotic diarrhoea, lactose intolerance, and allergies. Several studies have demonstrated the health benefits of prebiotics, particularly in positively modulating the gut microbiota and inflammatory response in patients with inflammatory bowel diseases such as IBD, ulcerative colitis (UC), or Crohn’s disease [123]. In several human studies, oral administration of multi-strain probiotic formulations containing well-characterised species of *Lactobacillus*, *Bifidobacterium,* and other beneficial bacteria has been associated with reduced disease activity, prolonged remission, and improved clinical symptoms [124]. In a study of patients with chronic colon inflammation, supplementation with a probiotic mixture led to a significant reduction in relapse rates compared with the control diet. These results were due to the ability of probiotics to maintain the balance of the intestinal microbiota, reduce mucosal inflammation, and promote intestinal barrier function [125]. Another study showed that a probiotic formulation improved clinical symptoms and inflammatory markers. This study also demonstrated higher remission rates than placebo, supporting its potential role as an adjunct in the management of chronic bowel inflammation [126]. In particular, *Lactobacillus* has been extensively studied and its use in the treatment of UC has been recognised, decreasing inflammation and oxidative stress and enhancing antioxidant defences [127,128].

The potential application of probiotics is not restricted to the treatment of GI tract disorders but is expanding to include the treatment of other disorders such as respiratory, cardiovascular, and central nervous system disease [122,129].

For both humans and animals, laws and regulations have established safety and quality criteria for probiotic substances, in particular, in the European Union, probiotics, fall under the regulation of the General Food Law and the claims regarding nutritional and health effects of probiotics are regulated by the Health and Nutrition Claims Directive (EC) no. 1924/2006 [130]. Also in animal nutrition the regulation (EC), No 1831/2003 establishes the rules governing the Community authorisation of additives for use in feed, including probiotic substances [131].

### Probiotics in Pigs: A Model for Human Health

Probiotics supplementation was mainly studied using an HMA piglets model. In particular, this approach was necessary in view of some significant differences in probiotics between the human and the animal model. The main difference is the species-specificity of the probiotics, which makes it necessary to choose species-specific strains for the target species selected for the study [132]. Furthermore, the human probiotic *Bifidobacterium* spp. is scarce or undetectable in the enteric tract of animal models such as the pig [133]. From this perspective, the use of HMA pigs makes it easier and more correct to evaluate the results obtained for human health. There is also the possibility of using challenge animals (vaccine or oral virus) to evaluate the effects of probiotics in a disease state. A literature search was carried out in CAB Abstracts and PubMed to identify articles published between 2005 and 2025. Further articles were identified by examining the reference lists of these articles. Results were limited to trials in these ranges of the following parameters: use of pigs as human models (GN or HMA), dietary supplementation with probiotics, as well as healthy or infected animals.

Two studies in pigs reported the use of a dietary multistrain probiotic blend proposed for humans (100 mg/kg of body weight for 112 d), containing *Streptococcus*, *Bifidobacterium,* and *Lactobacillus* spp. (SLAB51^TM^, Mendes SA, Lugano, Switzerland), and evidenced that dietary supplementation was able to affect goblet-cell secreted mucins and faecal *Lactobacillus* concentration, enhancing nutrient utilisation and growth [134,135].

All the other experimental trials were performed in GN piglets or HMA gnotobiotic piglets supplemented with probiotics and challenged with oral vaccine or virus, as reported in Table 3. The present data highlight that *Lactobacillus rhamnosus GG* and *Lactobacillus acidophilus* are able to reduce rotavirus diarrhoea symptoms and enhance the immune system for their use as an adjuvant in the vaccine. In fact, it is observed that supplementation with *Lactobacillus* spp. prevent the dysbiosis and have partial protective effects on the tight junction of protein expression caused by rotavirus infection.

In the review of [136] it is reported that *Lactobacillus rhamnosus GG*, one of the most commonly used probiotic strains in humans In fact, it has been reported to adhere to intestinal mucus and persist in the descending colon. Moreover, in vitro studies have reported the *Lactobacillus rhamnosus GG* efficacy against the viability, adherence, or infection of GI tract pathogens. This probiotic has also been used for the prevention and treatment of GI tract infections and diarrhoea, although its efficacy in humans has not been consistently demonstrated.

It is reported that in humans *Lactobacillus acidophilus* has antimicrobial activity against a wide range of pathogenic bacteria, and its immunomodulatory properties can initiate anti-inflammatory responses, enhance phagocytosis, induce defensin production and modulate intestinal permeability and restore balance of the gut microbiota, as reviewed by Liu et al. 2024 [137].

**Table 3:** Experimental studies on probiotics oral administration in gnotobiotic pigs utilising as a human model.

Probiotic Supplement	Dosage	Length (d)	Animals	Effects in GI Tract	References
*Lactobacilli acidophilus*	14 doses, up to 10^9^ CFU/dose	14	HMA GN piglet AttHRV oral vaccination	↑ LGG counts in feaces and gut * ↓ diarrhoea length * ↑ faecal scores *	[138]
*Lactobacillus rhamnosus GG*	9 doses increasing from 10^3^ to 10^6^ CFU/day	9	HMA GN Piglet AttHRV oral vaccination (0–15–26 d) Challenge HRV (28–35 d)	↓ diarrhoea percentage (HRV) * ↓ diarrhoea length (HRV) * ↑ protection by AttHRV vaccine * ↑ faecal scores * ↑ rotavirus-specific antibody * ↑ ASC *	[139]
*Lactobacillus rhamnosus GG*	9–14 doses increasing from 10^3^ to 10^12^ CFU/day	9 14	GN piglet AttHRV oral vaccination (5–15 d) Challenge HRV (28 d)	↓ onset of diarrhoea * ↓ faecal virus shedding * ↑ mucin production *	[140]
*Lactobacillus rhamnosus GG*	10 doses increasing from 10^3^ to 10^9^ CFU/day	10	HMA GN piglet AttHRV oral vaccination (5–15 d) HRV (28 d)	↑ microbial communities * ↓ *Enterococcus* * ↑ *Streptococcus* *	[141]
*Lactobacillus rhamnosus GG + Lactobacillus acidophilus*	10 doses increasing from 10^3^ to 10^9^ CFU/day	10	HMA GN piglet AttHRV oral vaccination (5–15 d) HRV (28 d)	HMA GN piglet AttHRV oral vaccination (5–15 d) HRV (28 d)	[142]
*Lactobacillus rhamnosus GG*	from 10^3^ to 10^12^ CFU/day	10	GN Piglet Challenge HRV (9 d)	↓ Intestinal autophagy-related protein expression * ↓ Ileum inflammation *	[143]

d, days, GN, gnotobiotic; HMA, human microbiota associated; AttHVR: attenuated human rotavirus vaccine; ASC: antibody-secreting cells; HRV virulent human rotavirus; ↑ increase; ↓ decrease; * *p* < 0.05.

Overall, these data support that probiotic administration in gnotobiotic pigs is able to protect gut health and enhance immunity during viral infection. In addition, the HMA gnotobiotic resulted in a suitable animal model to verify the gut and systemic immune response and the microbiota composition following the challenge (vaccine or oral virus). These data need to be verified by clinical trials in humans in order to replicate the correct dosage and the length of administration. Moreover, future studies can address the development of probiotic genetically modified strains that have the potential to enhance their functional properties, improving gut health.

## 6. Future Perspective

We have therefore seen how the use of HMA pigs can be a valid model for studies on human health, but some gaps remain. Indeed, the need for standardised protocols must be stressed. The lack of such protocols limits the reproducibility of studies using HMA pigs and does not allow the objective and reproducible definition of microbiota transfer methods and monitoring parameters [144]. Furthermore, this review has extensively evaluated dietary supplementation with prebiotics and probiotics, especially in the short term. It would be appropriate to analyse these results by conducting long-term studies to assess their effects on host physiology, immune development, and disease susceptibility [145]. Future studies in animal models and humans should focus on the improvement of probiotic properties, probiotic genetically modified strains, and their viability. Moreover, the synergistic use of prebiotics, which can improve GI tract functionality, could be deepened. A valuable future perspective could also be an interdisciplinary approach. The use of porcine-human organoid systems could provide a complementary tool to validate the results obtained in the current studies. It is also necessary to develop specific regulations and ethical frameworks for interventions on the microbiota using porcine models, which would facilitate the analysis and comparison of the results obtained.

## 7. Conclusions

This review highlights the importance of the pig model for conducting studies on the efficacy of prebiotic and probiotic supplementation for human health. Indeed, pigs’ anatomical and physiological GI tract characteristics are similar to humans, making them an ideal model for research. As reported, the pig is considered a suitable model for testing the efficacy of prebiotics. However, due to some differences in microbiota composition between the two species, HMA gnotobiotic pigs, which have a microbiota much closer to that of humans, are used to test probiotics. Considering the amount of literature on the promising action of prebiotics and probiotics in positively influencing gut health in human and animal models, it is necessary to increase knowledge about their effectiveness in counteracting gastrointestinal disorders and pathologies. Furthermore, it provides evidence that future advancements in therapeutic methodologies employing prebiotics and probiotics to enhance intestinal and systemic immune responses, as well as microbiota composition subsequent to diverse challenges, will be achieved through the utilisation of a porcine model.

## Figures and Tables

**Table 1:** Relevance of pigs as animal models to human health and disease.

Features	References
Simple availability for clinical trials	[14,17,18]
Omnivore diet	[9,19]
Genome is similar to that of humans	[11,12]
Analogous to human anatomy and organ size	[20]
Analogous to human gastrointestinal physiology	[13,15,21]
Systemic and mucosal immune responses similar to humans	[22]
neurobiological similarities to humans	[13,23]
Closely similar to human disease processes	[7]
Molecular mechanism of taste perception	[13]
